# In vitro antifungal activity of farnesyltransferase inhibitors against clinical isolates of *Aspergillus* and *Candida*

**DOI:** 10.1186/1476-0711-12-37

**Published:** 2013-12-05

**Authors:** Jianjun Qiao, Peiping Gao, Xiaoling Jiang, Hong Fang

**Affiliations:** 1 Department of Dermatology, The First Affiliated Hospital, College of Medicine, Zhejiang University, No. 79, Qingchun Road, Hangzhou, Zhejiang Province 310003, China; 2 Department of pharmacy, Taian City Central Hospital, Taian, Shandong Province, People’s Republic of China

**Keywords:** Aspergillus, Candida, Farnesyltransferase inhibitors, Antifungal susceptibility testing

## Abstract

**Background:**

Protein farnesylation is an important tosttranslational modification in fungi. We evaluated the antifungal activity of two farnesyltransferase inhibitors against clinical isolates of *Aspergillus* and *Candida*.

**Methods:**

Disk diffusion assay and broth microdilution assay were used to determine the antifungal susceptibility of two farnesyltransferase inhibitors (manumycin A and tipifarnib) against clinical isolates of *Aspergillus* and *Candida*.

**Results:**

Disk diffusion assay demonstrated both agents had activity against *Aspergillus* and *Candida*. The minimal inhibitory concentration (MIC) ranges for manumycin A against *Aspergillus* and *Candida* were 200 to 400 μM and 13 to >25 μM, respectively. Unfortunately, the MIC were vastly higher than the concentrations that inhibit the proliferation and viability of mammalian cells. The MICs of tipifarnib against *Aspergillus* and *Candida* were >1600 μM.

**Conclusion:**

The outcome of present study showed that farnesyltransferase inhibitors have activity against *Aspergillus* and *Candida*. This suggests that farnesyltransferase may be used as anifungal target in designing and developing new drugs.

## Introduction

Opportunistic fungal infections, mainly caused by the mold *Aspergillus* and yeast *Candida*, are life-threatening in immunocompromised individuals [1,2]. The infections continue to be a serious medical concern and are treated by only a limited number of antifungals: polyenes, azoles, echinocandins, and antimetabolites (flucytosine) [3]. Although these drugs are now being used in the prophylaxis and treatment of invasive fungal infection, the number of deaths due to invasive fungal infections remains high. Furthermore antifungal resistance and drug toxicity often limit the use of these drugs [3-5]. Therefore, it is needed to discover novel and effective antifungal agents to improve the prognosis of invasive fungal infections.

Farnesylation is a posttranslational process that occurs by formation of cysteine thioethers with farnesyl at or near the C-terminus. Farnesyltransferase is a ubiquitous enzyme that catalyzes posttranslational lipidation of the C terminus of more than 60 important signaling proteins in mammalian cells [6]. Several farnesyltransferase inhibitors have been successfully developed as anticancer drugs [6].

In human pathogenic fungi, homologues of farnesyltransferase were identified [7]. It has been reported that farnesyltransferase inhibitors result in growth reduction of *Cryptococcus neoformans*[8]. Deletion of genes encoding farnesyltransferase subunit leads to death of *C. neoformans* and *Candida albicans* and growth defect in *C. glabrata*[9-11]. Therefore, this enzyme may be a new target for the development of antifungal agents. In this study, we find that farnesyltransferase inhibitors have in vitro antifungal activity against clinical isolates of *Aspergillus* and *Candida*, two important human opportunistic pathogenic fungi. Results of this study may contribute to the development of new antifungals.

## Materials and methods

### Reagents and antifungals

**Figure 1:**
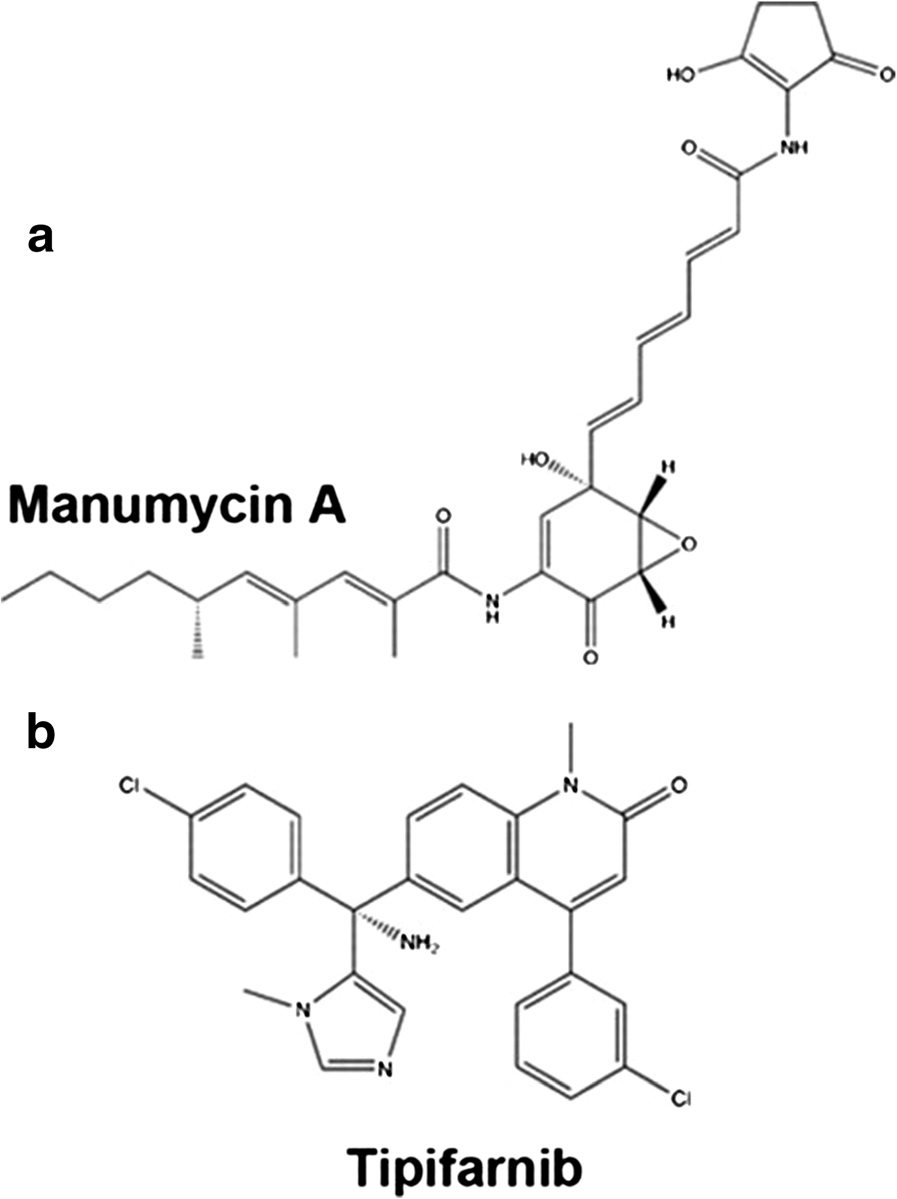
**Molecular structures of (a) Manumycin A and (b) tipifarnib.** Although both of the chemicals have anti-farnesylation activity, there molecular structures are quite different.

The farnesyltransferase inhibitors (Figure 1) manumycin A (Enzo Life Science International, Inc., USA; molecular weight, 550.7) and tipifarnib (Selleck Chemicals LLC, USA; molecular weight, 489.4) were dissolved in 100% dimethyl sulphoxide. Reference powders of itraconazole, voriconazole, caspofungin, and amphotericin B were provided by their respective manufacturers.

### Clinical fungal isolates

Clinical fungal isolates used for antifungal susceptibility in this study were obtained from patients in The First Affiliated Hospital, College of Medicine, Zhejiang University.

### Disk diffusion assay

Disk diffusion assay was performed as described previously [5]. A 200 μl suspension of each tested strain (1 × 10^6^ conidia/ml) was spread uniformly onto minimal medium plates. Blank paper disks 6-mm in diameter were impregnated with 10 μl (20 mM) manumycin A or tipifarnib. After drying, they were placed onto the center of inoculated agar plates. The plates were then incubated at 35°C, and the diameters of the zones of inhibition were measured at 48 h.

### Broth microdilution assay

The broth microdilution assay was done in accordance with the Clinical and Laboratory Standards Institute (CLSI) guidelines for filamentous fungi (CLSI document M38-A2) and yeasts (CLSI document M27-A3) [12,13]. Stock solutions were prepared in RPMI 1640 (Sigma) plates buffered to a pH of 7.0 with 0.165 M 3-(N-morpholino) propanesulfonic acid (Sigma). The range of final drug concentrations was 16 to 0.03 g/ml for itraconazole, voriconazole, caspofungin and amphotericin B, and 1600 to 3 μM for manumycin A and tipifarnib. The final inoculum was 0.4 × 10^4^ to 5 × 10^4^ cfu/mL for *Aspergillus* and 0.5 × 10^3^ to 2.5 × 10^3^ cfu/mL for *Candida*. The MIC endpoints for manumycin A, tipifarnib, itraconazole, voriconazole, and caspofungin were designated as the lowest drug concentration that prevented any discernible growth (complete growth inhibition) for filamentous fungi or the first well that demonstrated a prominent reduction in growth (a 50% reduction relative to that for the growth control) for yeasts at 48 h of incubation at 35°C. The MIC endpoints for amphotericin B were defined as the lowest drug concentration that completely inhibited fungal growth.

*C. parapsilosis* ATCC 22019 were included in each susceptibility test for quality control and assessment of reproducibility testing [12,13]. Each assay was performed in triplicate on three different days.

## Results

**Figure 2:**
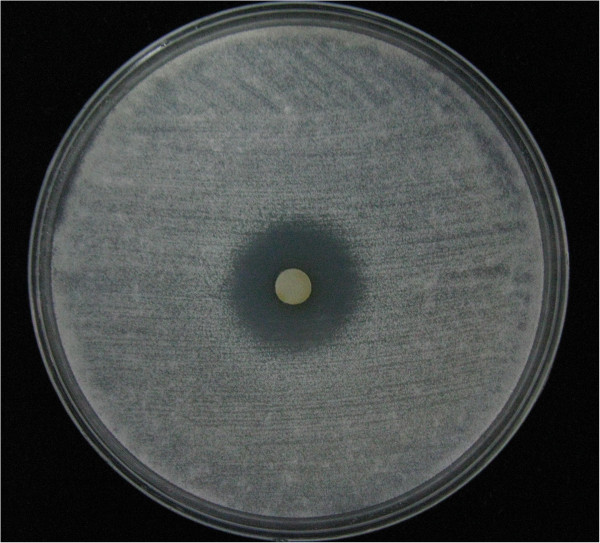
**Representative effect of manumycin A on the growth of *Aspergillus fumigatus*.** 2 × 10^5^ conidia of A. fumigatus-1 in 200 μl suspension were spread uniformly onto minimal medium agar plate. A 6-mm-diameter paper disk impregnated with 10 μl (20 mM) manumycin A was placed onto the center of the inoculated agar plate. The plate was then incubated at 35°C, and the diameter of the zone of inhibition was measured at 48 h.

Disk diffusion assay results showed that manumycin A produced zones of growth inhibition against the *A. fumigatus* isolates at 48 h of incubation, demonstrating the inherent activities of manumycin A against *A. fumigatus* (Figure 2). Disk diffusion assay also demonstrated that this drug inhibited growth of *A. flavus*, *A. terreus,* and *C. albicans*. The antifungal activity of manumycin A was further determined by broth microdilution assay, which showed that the MICs of manumycin A to 6 isolates of *Aspergillus* were 200 to 400 μM and those of 11 isolates of *Candida* were 13 to 25 μM (Table 1). However disk diffusion assay showed that tipifarnib, another farnesyltransferase inhibitor produced unclear zones of growth inhibition against both *Aspergillus* and *Candida*. Broth microdilution method showed that the MICs of tipifarnib to both *Candida* and *Aspergillus* were >1600 μM (Table 1).

**Table 1:** **Minimal inhibitory concentrations (MICs) of manumycin A, amphotericin B, itraconazole, and voriconazole against *Aspergillus *** **and *Candida *** **determined by standard broth microdilution method**

	**Amphotericin B**	**Itroconazole**	**Voriconazole**	**Caspofungin**	**Manumycin A**
	**(μg/ml)**	**(μg/ml)**	**(μg/ml)**	**(μg/ml)**	**(μM)**
*A. fumigatus*-1	1	0.5	0.5	0.06	200
*A. fumigatus*-2	1	0.5	1	0.06	400
*A. fumigatus*-3	1	0.25	0.5	0.06	200
*A. flavus*-1	1	0.5	1	0.125	200
*A. flavus*-2	1	0.5	1	0.125	200
*A. terreus*-1	2	0.5	0.5	0.06	200
*C. albicans*-1	0.5	0.25	0.25	1	25
*C. albicans*-2	1	0.25	0.5	0.5	13
*C. albicans*-3	1	0.5	0.5	0.5	13
*C. albicans*-4	0.5	0.25	0.25	1	25
*C. tropicalis*-1	1	0.125	0.125	0.5	13
*C. tropicalis*-2	1	0.25	0.125	0.5	25
*C. glabrata*-1	1	0.5	0.25	1	25
*C. glabrata*-2	1	0.25	0.125	1	25
*C. krusei*-1	0.5	1	0.5	0.25	25
*C. krusei*-2	0.5	1	0.5	0.25	25
*C. parapsilosis*	0.5	0.25	0.06	1	25

## Discussion

Recently, it had been reported that farnesyltransferase inhibitors showed antifungal activity against *Cryptococcus*[8]*.* In the present study, we showed that mamumycin A can inhibit growth of *Aspergillus* and *Candida.* To our knowledge, this is the first report of the in vitro antifungal activity of manumycin A against *Aspergillus* and *Candida*.

The antifungal activity of farnesyltransferase inhibitors suggests that inhibiting or deleting *RAM1* and *RAM2* genes should result significant defect of fungal growth [8,9]. We also find that deletion of *RAM1* gene in *A. fumigatus* results in significant growth defect of this fungus (unpublished data). Therefore fungal *RAM1* and *RAM2* may be a new target for design antifungal drugs. Although the MICs of the current farnesyltransferase inhibitors to *Aspergillus* and *Candida* were vastly high, we postulate that designing new farnesyltransferase inhibitors having high inhibiting effect on fungal farnesyltransferase is promising.

Both manumycin A and tipifarnib are farnesyltransferase inhibitors, however their chemical structures are quite different (Figure 1). This could explain why the MICs of the two agents against fungi are quite different.

## Conclusion

Our study showed that farnesyltransferase inhibitors have activity against *Aspergillus* and *Candida*. This suggests that farnesyltransferase may be used as anifungal target in designing and developing new drugs.

## Competing interests

The authors declare that they have no competing interests.

## Authors’ contributions

JQ and PG conducted laboratory analysis and drafted the manuscript. XJ and HF participated in the design of the study. All authors were involved in the interpretation of the data and approved the final manuscript.
